# Promoting networks between evidence-based medicine and values-based medicine in continuing medical education

**DOI:** 10.1186/1741-7015-11-39

**Published:** 2013-02-15

**Authors:** Myriam M Altamirano-Bustamante, Nelly F Altamirano-Bustamante, Alberto Lifshitz, Ignacio Mora-Magaña, Adalberto de Hoyos, María Teresa Ávila-Osorio, Silvia Quintana-Vargas, Jorge A Aguirre, Jorge Méndez, Chiharu Murata, Rodrigo Nava-Diosdado, Oscar Martínez-González, Elisa Calleja, Raúl Vargas, Juan Manuel Mejía-Arangure, Araceli Cortez-Domínguez, Fernand Vedrenne-Gutiérrez, Perla Sueiras, Juan Garduño, Sergio Islas-Andrade, Fabio Salamanca, Jesús Kumate-Rodríguez, Alejandro Reyes-Fuentes

**Affiliations:** 1 Grupo Transfuncional en Etica Clínica, Centro Médico Nacional Siglo XXI, IMSS, Av. Cuauhtémoc 330, Doctores, Mexico 06720 D.F., Mexico; 2 Unidad de Investigación de Enfermedades Metabólicas, Centro Médico Nacional Siglo XXI, IMSS, Av. Cuauhtémoc 330, Doctores, Mexico 06720 D.F., Mexico; 3 Instituto Nacional de Pediatría, Secretaría de Salud, Insurgentes Sur 3700, Insurgentes Cuicuilco, Mexico 04530 D.F., Mexico; 4 Unidad de Educación, Investigación y Políticas de Salud, IMSS, Av. Cuauhtémoc 330, Doctores, Mexico 06720 D.F., Mexico; 5 FES Acatlán, Universidad Nacional Autónoma de México, Av. San Juan Totoltepec, SN, Sta. Cruz Acatlán, 53150, Naucalpan de Juárez, Estado de Mexico, Mexico; 6 Instituto de Salud Pública del Estado de Guanajuato, Tamazuca 4, Centro 36000, Guanajuato, Mexico; 7 Universidad Anáhuac, México Norte, Av. Universidad Anáhuac 46, Lomas Anáhuac, Huixquilucan 52786, Estado de Mexico, Mexico; 8 Unidad de Investigación en Epidemiología, Centro Médico Nacional Siglo XXI, IMSS, Av. Cuauhtémoc 330, Doctores, Mexico 06720 D.F., Mexico; 9 Hospital Infantil de México "Federico Gómez", Secretaría de Salud, Dr. Márquez 162, Doctores, Mexico 06720, D.F., Mexico; 10 Fundación IMSS, Paseo de la Reforma 476, Mexico 06600, D. F., Mexico

**Keywords:** clinical ethics, values, continuing medical education, concurrent triangulation strategy, axiology

## Abstract

**Background:**

In recent years, medical practice has followed two different paradigms: evidence-based medicine (EBM) and values-based medicine (VBM). There is an urgent need to promote medical education that strengthens the relationship between these two paradigms. This work is designed to establish the foundations for a continuing medical education (CME) program aimed at encouraging the dialogue between EBM and VBM by determining the values relevant to everyday medical activities.

**Methods:**

A quasi-experimental, observational, comparative, prospective and qualitative study was conducted by analyzing through a concurrent triangulation strategy the correlation between healthcare personnel-patient relationship, healthcare personnel's life history, and ethical judgments regarding dilemmas that arise in daily clinical practice.

In 2009, healthcare personnel working in Mexico were invited to participate in a free, online clinical ethics course. Each participant responded to a set of online survey instruments before and after the CME program. Face-to-face semi-structured interviews were conducted with healthcare personnel, focusing on their views and representations of clinical practice.

**Results:**

The healthcare personnel's core values were honesty and respect. There were significant differences in the clinical practice axiology before and after the course (*P *<0.001); notably, autonomy climbed from the 10^th ^(order mean (OM) = 8.00) to the 3^rd ^position (OM = 5.86). In ethical discernment, the CME program had an impact on autonomy (*P *≤0.0001). Utilitarian autonomy was reinforced in the participants (*P *≤0.0001). Regarding work values, significant differences due to the CME intervention were found in openness to change (OC) (*P *<0.000), self-transcendence (ST) (*P *<0.001), and self-enhancement (SE) (*P *<0.019). Predominant values in life history, ethical discernment and healthcare personnel-patient relation were beneficence, respect and compassion, respectively.

**Conclusions:**

The healthcare personnel participating in a CME intervention in clinical ethics improved high-order values: Openness to change (OC) and Self Transcendence (ST), which are essential to fulfilling the healing ends of medicine. The CME intervention strengthened the role of educators and advisors with respect to healthcare personnel. The ethical values developed by healthcare professionals arise from their life history and their professional formation.

## Background

In the 21^st ^century, medicine tends to be dominated by two paradigms, evidence-based medicine and values-based medicine (EBM-VBM), which directly impact clinical decision-making processes in daily healthcare practice^a ^[1-6].

Modern biomedical science faces the challenge of reinforcing the pairing of EBM-VBM and constructing links and networks between them [7,8]. Continuing medical education (CME) promotes career-long competence with respect to medical advances (EBM); moreover, it can support fine-tuning of professional values and principles (VBM) [1,2,4,5,7,9-12].

Values are normative guidelines that allow us to consider actions, objects or situations as good, desirable, pleasant, convenient or useful towards certain aims [13]. These aims and the values that guide us towards them lend a mindful sensibility to our life and our professional practice [14]. Clinical practice is axiologically^b ^complex because it is not limited to describing, explaining or predicting what takes place within the human body (epistemological values: EBM), but it also acts on the bio-psycho-social spheres of a person and relates to his/her dignity [15,16] (social, political and ethical values: VBM). Furthermore, biomedical technical qualities are as important to healthcare as ethical qualities, yet ethical qualities are not always empirically evaluated. Emerging actions, devices and technical/scientific biomedical scenarios present increasing uncertainty and pose exponential risks that underscore the necessity of promoting an analytical-empirical axiology that places practice along a horizon of wisdom [7,17-22].

The healthcare sector is currently facing a crisis of knowledge, compassion, care, cost and values in general; however, few programs have addressed values among healthcare personnel, and little data exist concerning the effectiveness of such programs [23-27]. Values have a strong impact on the decision-making process and the final course of actions [27]. In other words, patients complain more about the lack of courtesy, warmth, understanding, care and communication than about the lack of updated attention protocols.

**Figure 1:**
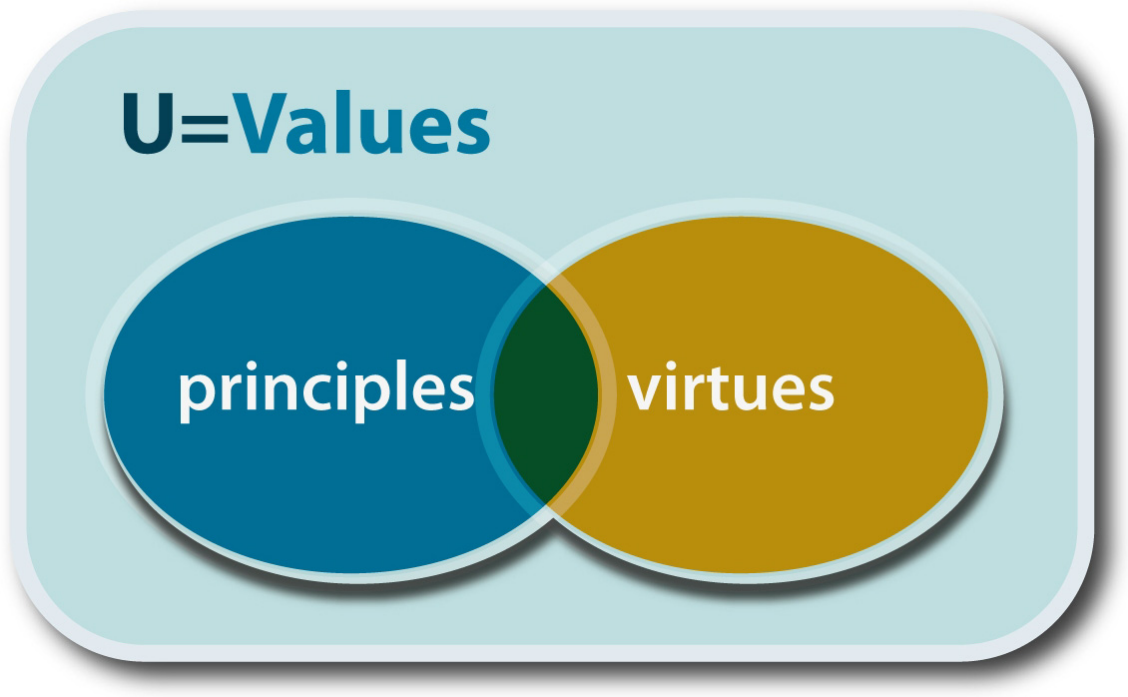
**A Venn-Euler diagram of values**. Values are the universe, while virtues and principles are subsets. The subset of virtue considers those values that refer directly to the healthcare personnel, their traits of character and decision-making. The subset of Principles expresses a normative procedure according to which actions can be guided to reach certain values [29].

Values are favorable dispositions towards aims that are sought. A physician is willing to act in accordance with the ends of medicine (healing, curing and caring) because they guide and give sense to his/her practice. These ends in medicine have traditionally been traced by clinical ethics in the form of principles and virtues. Principles state the deontological obligations of healthcare personnel and aim to offer an answer to ethical dilemmas. Principles will always be grounded on values. Principles explicitly state the values that we consider important [28], they express a normative procedure according to which actions can be guided to reach these values. [29]. Virtue ethics have resolved some of the shortcomings of principlism by arguing for the importance of the character traits and decision-making in moral discussions. If we think of a Venn-Euler diagram, values are the universe, while virtues and principles are subsets. That is, every virtue is a value, but not every value is a virtue; and the same goes for the principles; they are the expression of a normative procedure that is grounded on values, but at the same time they are valuable themselves (Figure 1). However, values have a broader focus, and they encompass virtues and principles alongside other objective goods that must be considered in ethical discernment (Figure 1).

Life preservation is a value that, in order to be upheld, is supported by several epistemic and ethical virtues and principles. Virtues such as wisdom, temperance and compassion aim at life preservation. In addition, principles such as beneficence are important in preserving life because they specify the obligations and provide explicit guidance to the agents' actions. However, in this paper, we maintain a broad perspective regarding values that allows us to move between virtues and principles and to consider the personal and social dimensions of patients and healthcare personnel in addition to the states of affairs that are valuable in strengthening the convergence of EBM-VBM (Figure 1). We acknowledge the great influence of virtue ethics and the principles of biomedical ethics, but it is our contention that a general and wider analysis can be carried out. Beauchamp and Childress' principles, in fact, express a normative procedure to uphold several values. For example, respect for autonomy demands action on behalf of the physicians towards an agent with the right to hold views and make choices based on personal values and beliefs. This principle specifies the actions to be carried out by someone seeking to uphold the value of autonomy. Therefore, our analysis will consider values as including principles and virtues (Figure 1).

As stated earlier, one of the ways to create a novel ethical environment is through education in clinical ethics. The first step is to carry out a situational diagnosis of what the ends of healthcare are considered to be, along with the values linked to these ends [7,14]. It is thought that when healthcare professionals are aware of these values, they will be guided by more careful reflection to adequately approach the dilemmas that arise [1,2,4,5,7,9-12]. Such reasoning has led us to pursue concurrent triangulation approaches that use quantitative methods to assess expressed values in medical practice and the decisions made when facing three clinical vignettes that pose ethical dilemmas. While capturing what health workers claim to be their values, we use qualitative anthropological methods and ethnography to define the values they display in their daily activities. Thus, we are able to analyze the concordance of such factors as life history, the doctor-patient relationship and ethical judgements regarding dilemmas that arise in the clinical practice.

The study's central hypothesis is that a cross-functional clinical ethics course is able to amalgamate EBM and VBM. Therefore, we seek to address the following questions: (1) What are the values of a group of healthcare professionals participating in CME in clinical ethics? (2) Why and how do participants respond to values in their clinical practice? (3) How does CME in clinical ethics impact the pairing of EBM-VBM? Here, we describe the successful use of CME to engineer networks between EBM and VBM.

## Methods

### Study design

**Figure 2:**
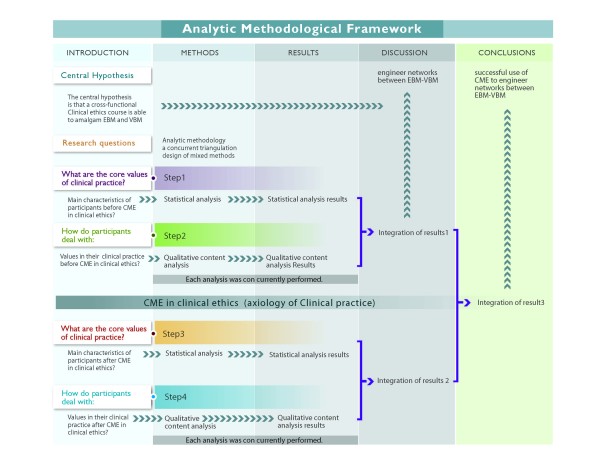
**Framework of the analytic methodology**. In step 1, we used statistical analyses to examine the axiology of clinical practice (values in healthcare, work values, virtues in medical practice and three clinical vignettes posing ethical dilemmas) and the characteristics of participants prior to conducting the CME in clinical ethics. In step 2, we used qualitative content analysis of semi-structured interviews (SSIs) to examine reasons for values usage in clinical practice and axiology in the ethical discernment process prior to conducting the CME in clinical ethics. After quantitative and qualitative research questions were examined, these results were integrated based on the mutual validation model, which regards the search for convergent findings as validity indicators as the most important purpose of triangulation. We explored potentially strong connections between EBM and VBM using qualitative results, while we inferred the extent of the benefits of novel networks using quantitative results. After conducting the CME in clinical ethics intervention, we repeated the analyses (steps 3 and 4), and the full results were integrated.

A concurrent triangulation design of mixed methods' strategies to analyze both quantitative and qualitative data was used to empirically explore the axiology in the clinical practice of Mexican healthcare professionals [30], as illustrated in Figure 2. Mixed methods were combined for complementarity, where each method addressed a different aspect of the research questions and highlighted new connections [31]. Quantitative methods were used to determine the self-declared values of clinical practice before and after the CME program. The qualitative semi-structured interviews (SSIs) and three clinical vignettes (CVs) were employed to explore participants' experiences and representation of their clinical practice, with an emphasis on experiences with the patient-healthcare personnel relationship and ethical discernment. We conducted a quasi-experimental, observational, longitudinal, comparative and prospective study that allowed us to describe the state of the art and to strengthen practices favorable to the pairing of EBM and VBM (Figure 2).

### Study population

Mexican healthcare personnel with current active practices in several clinical medical areas were invited to participate in an online course in clinical ethics, at no cost, from September 2009 to February 2010. Registration was conducted during a two-month period prior to the CME program (N = 2,891). During registration, each participant provided his/her demographic data and responded to an online survey (Table 1).

**Table 1:** Sociodemographic characteristics at two stages

Variable		Stages	
		**Registration (n = 2,891)**	**Final (n = 973)**

**Age**	Mean ± (SD)	39.38 (±9.9)	38.2 (±9.7)
**Sex**	Female	62%	70%
	Male	38%	30%
	First	41%	37%
**Level of**	Second	32%	35%
**Healthcare**	Third	22%	19%
	Central	5%	9%

Healthcare professionals who enrolled in the online course represented every Mexican state and organizational level of healthcare. The primary healthcare level is preventive and family medicine. The secondary level comprises different medical specialties and general surgery. The tertiary level includes highly specialized medical attention.

The research ethics committee of the Mexican Institute of Social Security (IMSS) approved the study. All participants received written and oral study information and signed a letter of informed consent granting the authors permission to use and publish the data and results of this study.

### CME in clinical ethics Intervention

The course was designed by a cross-functional group (including medical doctors, teachers, anthropologists, sociologists, philosophers and bioethicists), and it included five modules: the person and human dignity, medical ethics, healthcare professional/patient relationship, clinical ethics committees, and methodologies for ethical discernment.

The Anahuac University and the IMSS awarded those who completed the course with a 60-hour CME certification. This on-line course provided information to healthcare personnel about ethical terms, concepts and theories. Additionally, the course reviewed guidelines for ethical decision-making, which included exploration of personal values in addition to problem-solving exercises (patient simulation, motivational videos and online discussion forum) regarding how to apply ethical concepts and theories to ethical dilemmas (Figure 2).

## Quantitative study

### Instrument design (survey)

**Figure 3:**
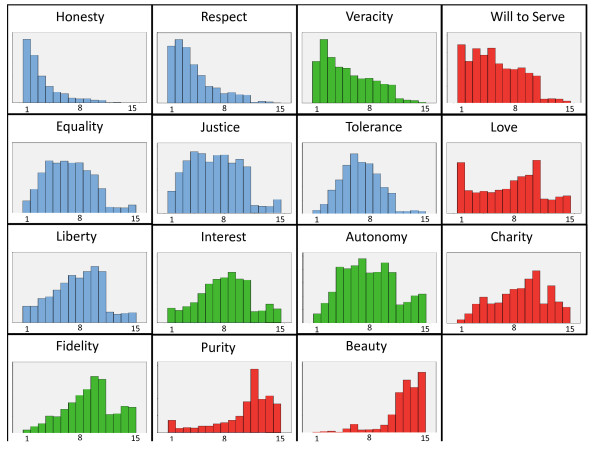
**Hierarchy of values in clinical practice in Mexico**. Each of the charts shows the state of values before the intervention (*n *= 2,891). Deontological values are in blue, aretological values are in red, and utilitarian values are in green. The lower the values, the higher their level of importance.

Although numerous survey instruments measuring values are used worldwide [15,16,32,33], they do not explore the values or virtues specific to medical practice (Figure 3). A set of survey instruments designed by a cross-functional team (an expert panel in clinical axiology) were drafted and initially tested on a small sample of 10 participants; they were subsequently used in this paper. These instruments were used to assess the respondents' values and priorities in healthcare practice before and after the CME program. The survey collected participants' declaration of personal values in addition to measuring their values with respect to healthcare, work and medical practice. The survey also included three clinical vignettes posing ethical dilemmas [34].

### Work values

To assess work values before and after the CME intervention, we used an instrument proposed by Schwartz that operationalizes four high-order values [33] in the work environment. These high-order values encompass a total of 16 items that constitute the EVAT (Escala de Valores hacia el Trabajo) scale [35]. The EVAT scale has been used among large samples of Mexican, Spanish, Portuguese and Italian workers [36].

### Ethical discernment instrument

The survey included three clinical vignettes that were used to assess discernment in some of the common ethical dilemmas encountered in medical practice: patient confidentiality, informed consent (autonomy) and withdrawal of care (beneficence). The clinical vignettes revealed differences in discernment before and after the CME [34].

### Statistical analysis

Distribution of participants according to gender, age, profession/discipline and healthcare level is described for each of the two phases (Table 1). We explored the before-and-after changes of self-declared values in medical practice. The changes in the before-and-after ranking of these items were determined using the Bonferroni-corrected Wilcoxon signed rank test. Differences among hierarchical items were determined using the Kruskal-Wallis test, followed by the Steel-Dwass test for pairwise comparison. Parametric tests (Student's *t*-test, and paired *t*-test) were used for the statistical analyses and were confirmed by non-parametric tests (Mann-Whitney *U*-test). For the cluster analyses, the mean and standardized values of the aretological, deontological and utilitarian groups were obtained. For the work values analyses, we obtained the means for the group of high order values: openness to change (OC), self-enhancement (SE), self-transcendence (ST) and conservation (CO). These values were illustrated on a three-dimensional scatter plot.

### Qualitative study (interview)

The aim of the qualitative component of the study was to explore the self-representations of the healthcare professionals. Purposive sampling was undertaken [37] to include a range of types of healthcare professionals and a range of types of health institutions. A semi-structured interview guide was designed by an expert panel on clinical axiology and pilot-tested by Nava Diosdado *et al*. [38]. Briefly, we designed an instrument that would allow us to identify values and to assess career goals in the following categories: life history, career goals, reasons the interviewee joined the profession, working values, how the interviewee resolves his or her day-to-day problems, type of doctor-patient relationships established, projections of the interviewee on his or her patients, attitude towards clinical ethics, and how interviewees see themselves in the next 10 years.

Face-to-face semi-structured interviews were conducted with healthcare personnel by anthropologists. The interviews focused on the personnel's views and representations of clinical practice. The interviews took place between September 2009 and February 2010, before the CME intervention (78 interviews) and after the CME intervention (42 interviews). Interviews lasted approximately 80 minutes and were recorded and later transcribed.

The transcriptions of interviews were analyzed using the content and thematic analysis method described by De Hoyos *et al*.^c^. Briefly, all data were analyzed following five steps: familiarization with the data through listening and immersion in the raw data several times, identification of a framework, coding, charting and interpretation. An axiological framework was developed in line with Schwartz's work values and Pellegrino and Oakley and Cocking's findings regarding the virtues and vices of healthcare professionals. The main emerging themes were representations of medical practice. Beliefs, desires, meanings and their axiological interactions structure of clinical practice were studied. One hundred codes were grouped according to the following subjects: life history, workday, ethical discernment, patient-doctor relationship, medical procedures, decision making, ethics committee and future expectations of healthcare personnel.

For both sets of interviews, Atlas.ti 6.0 software^d ^was used to identify emergent themes in addition to the views of the participants. Each interview ended with the presentation of short vignettes concerning clinical cases. The use of vignettes with open-ended questions in qualitative research attempts to determine perceptions, attitudes and moral values, all of which are particularly pertinent for this study [31]. Codification and analysis were performed by the cross-functional team. The chart was thoroughly discussed and interpreted in meetings among all members of the cross-functional team (Figure 2).

### Data analysis for ethics

We used an inductive, ethics-based process of analysis, following Josep Lozano's classification of ethical theories founded on three major approaches [39,40]: the first one is virtue (or aretological) ethics, where goodness is determined in relation to the type of moral agent involved and the context of his/her actions. The second is deontological (that is, duty or rule) ethics, where the emphasis is placed on the generality of the rules and their rationality. Finally, there is utilitarian ethics, which identify the good with its consequences. The utilitarian principle demands the maximization of the good produced by one's actions.

These approaches allow the specification of which values are in conflict for a given ethical dilemma: the confrontation between virtue and duty, virtue and efficiency, or duty and efficiency. The cross-functional research team identified codes for units of meaning in the survey.

## Results

### Quantitative analyses

#### Study population

Axiological situational diagnostic data were obtained for 2,891 healthcare professionals who were initially enrolled in the course (registration). The final sample included 973 healthcare professionals who successfully completed the course and who had also completed the before-and-after survey instruments as well as the clinical vignettes. Table 1 shows the socio-demographic features of the healthcare personnel during the two stages of the study.

We confirmed that the 973 participants of this study represented the initial participants in the course, using demographic and professional survey information. No differences were found with respect to factors such as professional distribution, gender, age, organizational level of healthcare or geographic distribution.

Most of the participants were female (62%). The predominant profession was physician (57%), followed by nurse (20%). Participants' ages ranged from 17 to 82 years, with 43.5% of the participants being between 36 and 50 years of age (median age of 44 years). The healthcare personnel represented different regions of Mexico. The sample included the four organizational levels of Mexican healthcare: first level, 41%; second level, 32%; third level, 22%; and central level, 5%.

Of the participants, 40% had prior training in bioethics through courses taken during their higher education. Participants' primary motivation to enroll in the course was to improve their professional performance (54.6%), followed by the desire to increase their knowledge of clinical ethics (36.3%).

#### Axiology in clinical practice

**Figure 4:**
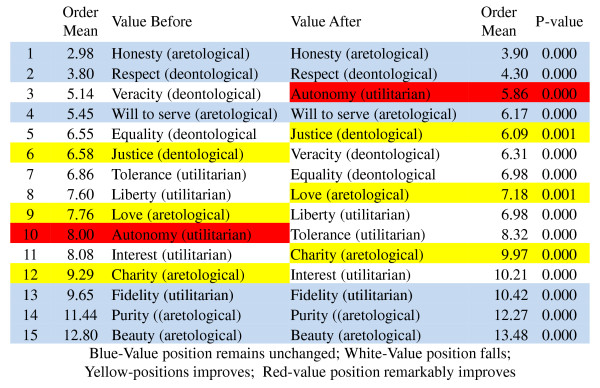
**Hierarchy of values in clinical practice before and after CME intervention**. All the values presented statistically significant change before and after the intervention (Wilcoxon Signed Rank Test with Bonferroni's correction).

The values of clinical practice refer to favorable attitudes, actions and situations that will help to achieve patients' wellbeing. The priorities and value rankings of 2,891 participants are shown in Figure 3. The values of clinical practice differed significantly before and after the educational intervention (Figure 4).

Healthcare professionals in Mexico assigned great importance to the values of 'honesty' and 'respect' by placing them first and second, respectively, in their hierarchy of values, whereas a low level of importance was given to values such as 'fidelity', 'purity' and 'beauty'. Notably, the two highest and the three lowest values in the ranking remained unchanged after the clinical ethics course (Figure 4).

The value of 'autonomy' was ranked high (third) by the participants after completing the course, whereas prior to the course, it was ranked 10^th ^(mean position shift from 8.00 to 5.86, a statistically significant difference) (Figure 4). Other values deserving attention were 'justice', 'liberty', 'love' and 'charity'; these values assumed higher hierarchical positions after the CME intervention (statistically significant, (Figure 4)).

**Figure 5:**
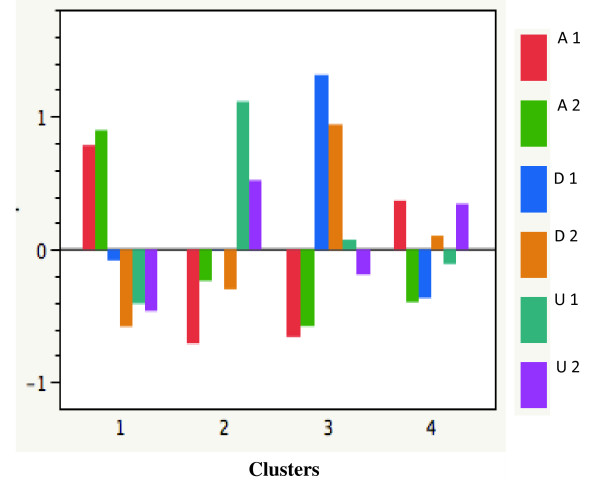
**Ethical clusters found among Mexican healthcare personnel**. Aretological values are A; deontological values are D; utilitarian values are U. Before (**1**) and after (**2**) the educational intervention.

Figure 5 shows the ethical clusters found in Mexican healthcare personnel based on Josep Lozano's classification of ethical theories (aretological, deontological and utilitarian) [39]. The first group is deontological/utilitarian; the second, aretological/deontological; the third, mainly aretological; and the fourth, aretological/utilitarian.

#### Ethical dilemmas and values

A dilemma is a situation in which a person may choose only one of two courses of action, each of which seems to be well supported by certain values. The survey encompassed three clinical vignettes [34]. We assessed the clinical vignettes following Gisondi's definitions of each value [34] in a representative sample of 448 participants. We assigned the maximal score to 'patient confidentiality' when "the healthcare professional does not discuss clinical or confidential information about a patient with others" [34] or only disclosed information in the case of risk to public health. Confidentiality was one of the strongest values for participants and was not modified by the educational intervention (*P *= 0.06). For 'beneficence', we assigned the highest score to situations where "healthcare personnel identify the surrogate decision maker, obtain paperwork for advance directives, withdraw care when appropriate, and when needed, requested some form of palliative care for a patient" [34]. This value was present among healthcare professionals working in Mexico and was not modified by the educational intervention (*P *= 0.1618).

The value of 'autonomy' was labelled as 'utilitarian autonomy' when healthcare personnel attempted to obtain informed consent or voluntary discharge, and 'deontological relationship-based autonomy' was a situation in which "the health personnel attempted to obtain informed consent from the patient or surrogate by explaining common risks, benefits, and alternatives (including no intervention), while querying for and answering patient questions in an unbiased fashion" [34]. Educational intervention had an impact on both types of autonomy (*P *≤0.0001). Utilitarian autonomy was largely reinforced in the participants (*P *≤0.0001).

#### Work values

**Figure 6:**
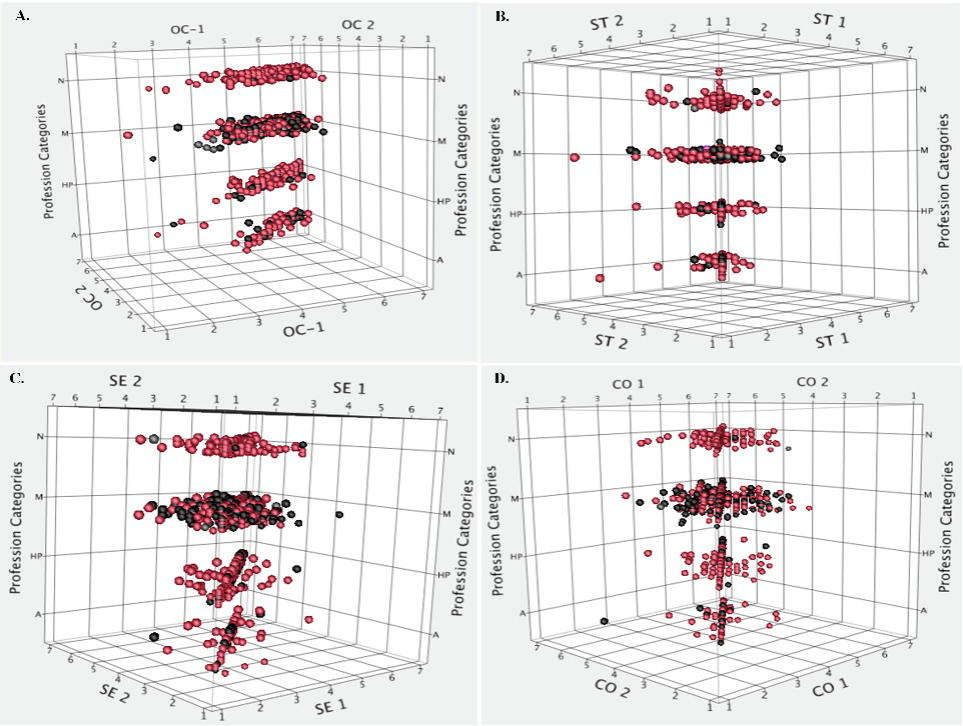
**Four high order values (Schwartz's theory) before and after CME intervention**. Each row includes N = Nurses, M = Medical Doctors, HP = Others Healthcare Professionals. A = Administrative personnel. Spheres in red are females. 1 = Before and 2 = After educational intervention. **A: **Scatter plot in 3D. Openness to Change. Spheres representing post-CME intervention appear compacted. **B: **Scatter plot in 3D. Self-transcendence. Spheres representing post-CME intervention appear compacted. **C: **Scatter plot in 3D. Self-Enhancement. **D: **Scatter plot in 3D. Conservation.

According to Schwartz [32], individual behavior is highly influenced by a specific set of values that the agent finds important. As observed in Table 2, the differences encountered between the means on openness to change (OC) and self-transcendence (ST) before and after CME intervention were highly significant (*P *<0.001) according to the values of the *t *tests. These results allow us to confirm our central hypothesis that we can engineer strong connections and networks between EBM and VBM through CME (Figure 6A-D). Individuals who assign a high priority to the high-order value of ST tend to be more satisfied in their jobs because they introspectively evaluate their professional values and find them to be aligned with the ends of medicine [41] (Figure 6A-D).

**Table 2:** Work values

	Median	**Mean ± S.D**.	Student's *tP*-value	C.I. 95%	Wilcoxon*P*-value
Openness to change-B	5.75	5.69 ± 0.89	0.000^c^	-0.09;-0.04	0.000^c^
Openness to change-A	6.00	5.77 ± 0.87			
Self-transcendence-B	6.25	5.99 ± 0.94	0.001^c^	-0.08;-0.006	0.010^c^
Self-transcendence-A	6.25	6.05 ± 0.89			
Self-enhancement-B	2.5	2.71 ± 1.10	0.019^c^	-0.07;-2.34	0.035^c^
Self-enhancement-A	2.75	2.75 ± 1.11			
Conservation-B	3.5	3.50 ± 0.89	0.22^d^	0.01; 0.05	0.171^d^
Conservation-A	3.5	3.48 ± 0.90			

B = before, A = after, ^c ^With significant differences, ^d ^Without significant differences.

### Qualitative content analysis

To strengthen the knowledge claims of the study, the declared values considered in the quantitative analysis were paired with a qualitative analysis that aimed to gain insights into the representations of the actors and their beliefs. A well-validated study on a subject such as clinical ethics and values would be incomplete without both quantitative and qualitative perspectives.

#### The rise of vocation and its consolidation

**Figure 7:**
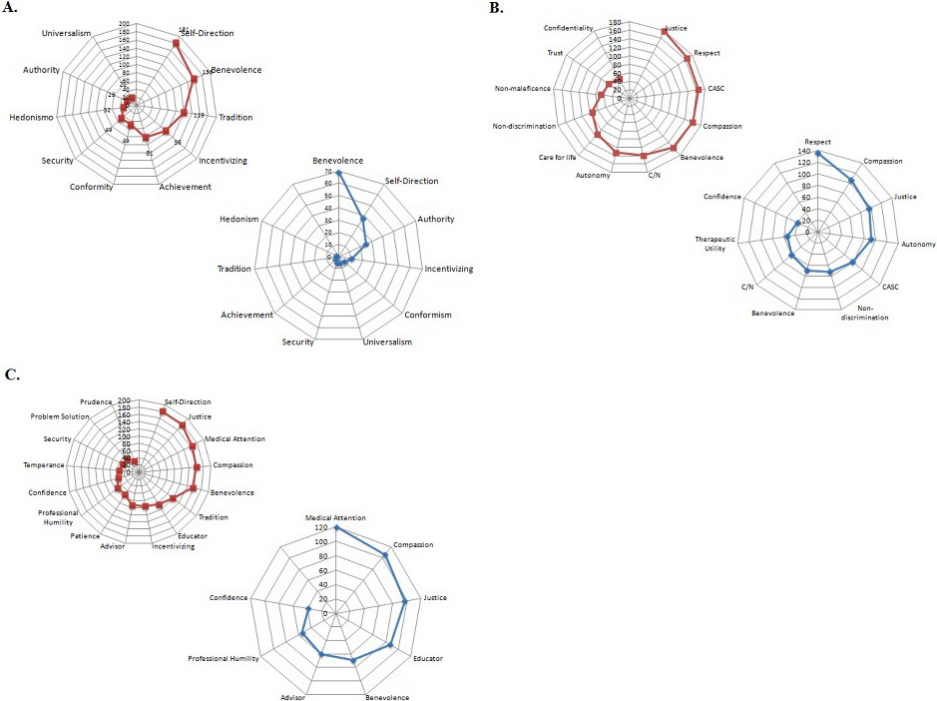
**Semantic networks**. Keywords were identified with Atlas.ti 6.0 software. Words were sorted according to the frequency of their appearance in the interviews. The cut-off point, which divides the set of words into a high-frequency and low-frequency groups, was identified. Radial graphs explaining the frequency of appearance were created with MS Excel 2007. Red indicates before CME, and blue indicates after CME. **A**: Semantic Networks for Life History. **B**: Semantic Networks for Ethical Discernment. **C**: Semantic Networks for Healthcare Personnel-Patient Relationship.

When qualitatively tracing the most salient values in the life history of the individuals interviewed, we found that healthcare personnel referred to a core of values, where beneficence is dominant together with tradition, incentivizing, achievement and conformity, and that those traits led them to choose a career in the medical field (Figure 7A). Self-transcendence (ST) is analytically divided into 'beneficence' as a concern for those with whom one is in contact. 'Universalism' is an abstract sense of goodwill with respect to the health of the general population. Although both values were included in the healthcare personnel's representations, beneficence was predominant (Figure 7A).

The configuration of beneficence as a guiding value for the vocation of these professionals is associated with two different and sometimes conflicting sources. In one, social relations are protected through conformity with established values and traditions; in the other, innovations in these values are made when incentives encourage new achievements in the medical field (Figure 7A).

The educational intervention permitted the rediscovery of certain values that would consolidate the vocation of the healthcare personnel; even if the participant's perspective shifted, the centrality of beneficence remained. Self-direction and incentivizing came to play an important role once the professionals acquired greater experience, and this experience supported improved knowledge and performance. While self-direction and incentivizing demonstrate an openness to change, they are still well balanced with the protection of social relationships through adherence to prevailing values (Figure 7A and Table 3).

**Table 3:** Values and healthcare personnel roles

Values and Healthcare Personnel Roles	Quote
**Justice**	*"Yes, I have treated homosexuals, and I relate to them normally."*
	*"You treat everyone the same, you give them [prisoners] the same attention as the rest. I mean, if you have to treat them, and you have to do it that way. I think that maybe it's a risk of the profession, but either way, it's the patient and you have to... treat everyone the same... equally." *
**Respect**	*"This has indeed happened. I have a patient with HIV, and ever since he first came to me, he told me, 'You know what? Neither my mother, nor my siblings know I am HIV positive, and I don't want them to find out'. Therefore, I believe you have to respect the patient's decision; if the patient is conscious, if he's oriented, you have to respect his ideology."*
	*"I'd say that in case I don't really need to transfuse him, I wouldn't do it, and not for my personal security or the patient's security, but because one tries to respect the parent's decision, but the first thing to do is to convince them to go ahead with it."*
**Compassion**	*"We had lots of friction with them. All the personnel had frictions with that family. Then, I talked to the wife, who felt awful because she could not get any money because she had already sold everything. I told her to draw close to any religious faith. It is as if you were changing somebody's life; a psychologist must intervene; something must be done to make them sensitive, to help them improve. It was not until I told the woman to start working that she told me she did not know how to do anything. However, I told her she needed to help her husband, and the man was not making any progress. It took her, like three months; the woman started working, and it was very hard for her."*
	*"I put myself in the patient's shoes. I have always said to my daughters and husband, if I come to this [need of resuscitation], I am against doing everything until the end; tubing him, giving him electric shocks in the heart. Tubing him all over is just tormenting the patient and his family, too. It is very hard to come and see your loved one with tubes all over; it's shocking. I have always said that I don't want that for me. I wouldn't do it either to someone else."*
**Benevolence**	*"I chose nursing. To tell the truth, ever since I was a kid, I liked the whole helping situation, being always aware that my brother was sick, and me being there, helping him. It's as if I always thought of having to be there, giving something to others."*
	*"One just doesn't [treat him]. And yes, we have been asked, and one just doesn't do anything. One waits for the end. You just stay there with him, but let me say we never leave them alone. They go into cardiac arrest and all, but they do tell you, "If something happens to me, I don't want to be tubed or anything like that", but you just stay there, with him. I mean, I don't know, you talk to them, maybe even pray or I don't know, but you just stay there."*
**Professional Humility**	*"Youngsters are forgetting that here in Mexico we practice a clinical medicine, using clinical skills. You must figure out many things. Just by simply exploring the patient, I can get a feeling that the problem might be this or that, and I support my opinions with lab tests in order to confirm or rule out anything. Now, it's going the other way. If a patient tells them they have a headache, they send them for a CAT scan and forget about the rest. That is why, nowadays, we are having so many problems. Because it's true; you can always send them for a CAT scan, but you neglect other skills."*
	*"Now, I have to accept that perhaps they want to take other opinions, visit other surgeons, or maybe they don't want to be operated on. Maybe there's another alternative, and one has to accept that."*
**Self-direction**	*"When a patient does not follow the treatment, I comply with the rules by telling them what they need to do. I tell them what their problem is because I cannot go to their place and take care of them. They are old enough, and they are responsible. I try to make the patients feel committed so that they don't blame the doctor."*
	*"I go to my colleagues at the ER, and I help them to set up an IV for a patient in pain. I just stay with my pregnant patients, or I go with my vaccination colleague and help her capture the data about the shots the patients are getting."*
**Compliance with standards**	*"That has also happened to me with certain religion that prohibits this. In this case, the family and the patient were told about the risks if he was not transfused, and in spite of everything, of the complications and all, they refused. One has to protect oneself, and you can ask them to sign their refusal of treatment, despite being informed, and that way one is a bit more protected. One tells them about the consequences; if they do not accept the procedure, it is their responsibility, one simply informs them."*
	*"In this case, we would need to talk about it [a do-not-resuscitate order] with the family and the patient, in writing and in accordance, if the patient is conscious and aware of what he says, in control of himself. We need him to be capable. Many people express their will during their lifetime, they make it evident for their families, it is the same for those who want to donate organs: 'You know what folks, if something happens to me, I want to donate my organs'. This is very frequent, and in this type of patient, if he expresses his will and is completely conscious, a document is written, and he signs it."*
**Capacity to appraise situations and consequences**	*"That has also happened to me with certain religion that prohibits this. In this case, the family and the patient were told about the risks if he was not transfused, and in spite of everything, of the complications and all, they refused. One has to protect oneself, and you can ask them to sign their refusal of treatment, despite being informed, and that way one is a bit more protected. One tells them about the consequences; if they do not accept the procedure, it is their responsibility, one simply informs them."*
	*"One thing that I perceive and admire from the haematologist, for instance, is that, with the patients with leukaemia, she tells them: 'This is the situation: your treatment is not working'. Then, they just remain there, thinking. But she gives them a choice. 'You have to decide; if the treatment is not working, they are going to ask you, if you are going to stay and continue with the treatment. In the meantime, you might pass away, you need to decide if you want to go home and enjoy your family'."*
**Tradition**	*"Family intervention is very important because it is the family who brings the patient; even if most of the times the patient comes by himself, the family is the most worried. The role of the family is very important because relatives are the ones who will follow the treatment at home. We only stabilise the patient."*
	*"For me, the family is essential in the treatment. [Family] is the centrepiece of society; very often the failure or success of the treatment depends on the family."*
**Advisor**	*"That's why I always try to play music in my practice, all kinds of music, but mainly instrumental music. It's just like they say, music calms the beasts because the beast can be unleashed at any moment. I try to calm down and think about what I am going to say. Tell him why things are going wrong: 'Things might get complicated with this and that'. For me, it has been useful to tell them they are not orders but suggestions or recommendations that I hope they follow. And if they don't want to follow them, I cannot take care of them. They are the ones who will end up worse. It won't be me or their families, but themselves. That works for me; that, I learned with time and with teachers."*
	*"I already enjoyed talking and listening, but now I have better reasons to do so! I do like talking to patients if they tell me about their family and personal stuff, as well as other things. One has very frequent patients; they come here all the time, and you already know them. You know their whole life and work; even their family knows you and identifies you. And well, yes, my attitude has changed. I am more communicative, more participative with my patients too.... For me, the most important thing this course has given me is that: more humility, more... one very important difference."*
**Educator**	*"I try to explain their disease to them and how we will proceed in the treatment because I wouldn't like it if someone left me with doubts. Sometimes, I see the doubt in their faces and I try to explain it to them; the same goes with the treatment. I believe we have different educational levels, and sometimes one talks to them with lots of technicalities. Before studying, we also didn't understand a thing, as if we spoke in another language."*
	*"I would make him see that it's necessary, that it's part of the treatment, that there are maybe risks associated to this surgical procedure because he can lose so much blood or have some other problems, and that therefore, it is necessary."*
**Medical Attention**	*"I treat everyone. Because I am a family doctor, I treat the child, the father, the mother, the insured, everything. On average, I treat approximately 24 patients a day. I spend approximately 15 minutes with each of them, but sometimes I can spend 5 minutes with one person and an hour with another. Sometimes there is not enough time. You sometimes need more time, but that is compensated for if another patient comes with something simpler."*
	*"They come and tell me: 'You know what? I went with that doctor, and he did not explain anything. I want to know what my problem is. I want to know what I have'. Then, I do research, I go through the medical records and files, I try to reach a conclusion, and well, I try to let the patient know about it."*

For each value and role, the boxes on top correspond to the interviews before CME intervention. The boxes at the bottom correspond to the interviews after CME.

#### Ethical discernment

Daily medical practice encounters ethical dilemmas when treatment efficiency and other related values must be taken into account to help the physician make a wise decision. The basal values that are most often cited in healthcare professionals' representations are justice, respect, medical attention, an ability to appraise situations and their consequences, compassion and beneficence, followed by compliance with norms and autonomy (Figure 7B).

Autonomy, beneficence, justice and non-maleficence are the values suggested by Beauchamp and Childress' principlism for ethical discernment. As stated in the background section, principles express normatively a procedure to uphold a value (Figure 1). In this case, we analyze directly the values that are reached through these principles. However, our data show that autonomy was not a founding value in the ethical-discernment for healthcare professionals prior to CME; rather, among these four values, justice is relevant and precedes the rest, and beneficence is second. Both values relate to ethical judgement (Table 3 and Figure 7B).

In the analysis, with the exception of justice, values such as respect and compassion were considered more relevant to ethical judgment than the remainder of the values related to principlism (Figure 7B). Respect and compassion demonstrate that the patient-doctor relationship is understood in different ways that aim to approach the patient with dignity. In general terms, the interviewees described a discernment that is justice-centered. (Table 3 and Figure 7B).

Additionally, we investigated the interviewees' representations of how medical attention should have an impact. Generally, the medical practitioner described not making decisions based on the notion of an ultimate end of the practice but rather on the ability to appraise specific situations. The ability to appraise situations is, of course, useful in maintaining life and avoiding risks, but it also helps to clarify courses of action in ethically complex cases (Table 3 and Figure 7B).

The educational intervention modified participants' configuration and hierarchy of values. After the CME intervention, values such as respect, compassion and justice were predominant over the others. Autonomy moved from the eighth position to the fourth position according to importance (Figure 7B).

#### Healthcare personnel-patient relationship

##### Different kinds of relationships between patients and healthcare personnel

We were able to discern a bundle of values in the specific relationships that we studied. The initial values that prevailed in this relationship were self-direction, justice, medical attention, compassion and beneficence. Again, we noticed that the values related to virtue ethics are stronger than those related to principlism (Figure 7C).

##### Medical attention, the main role played by healthcare professionals

In describing their functions, all interviewees quickly noted that their job was to provide medical attention; they were all immediate and precise in their description. The medical practitioners eloquently spoke about the different types of patient they meet; they recognized in advance the types of persons and diseases they would encounter (Table 3).

In relation to the particular situations in medical attention in which the link with the patient is the common denominator, we found that values such as compassion, justice and beneficence, self-effacement and trust are salient (Table 3 and Figure 7C).

The health professionals showed great patience when working with patients after the CME intervention. Issues that would normally act as a barrier between the health professional and the patient became an opportunity for the health professional to provide the patient with comprehensive medical care. The healthcare personnel stated that when patients abandon their treatment against medical advice, reactions such anger, disappointment or discomfort are not uncommon. Following the CME intervention, they resolve these issues by informing patients about the treatments and their benefits. Educating patients implies transmitting scientific information that provides them with some certitude regarding their treatment in general. This information must be provided in an understandable and non-condescending way (Table 3).

##### Virtues in clinical practice

**Figure 8:**
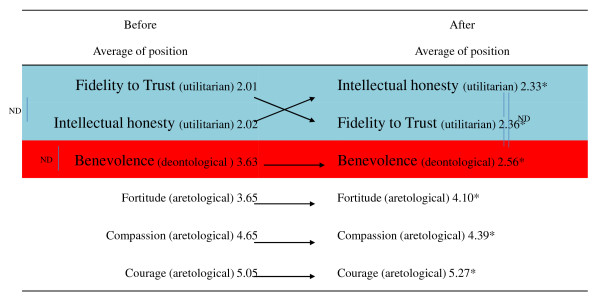
**Virtues of medical practice**. *Denotes a statistically significant difference before and after the intervention (Wilcoxon Signed Rank Test with Bonferroni correction). Vertical lines tie the pair of values between which no statistically significant difference was found (Steel-Dwass All Pairs Comparison).

One way of creating a novel ethical environment and improving the quality of medical care is fostering new values to face the challenges of clinical practice. Among the different values that are important for medicine, we find several virtues. Virtues are values that refer directly to the healthcare personnel, their traits of character and decision-making (Figure 1). Hence, special attention must be paid to the virtues that are fostered in clinical practice because well-established character traits will help practitioners make sense of their own practice and, at the same time, pursue the valuable ends of medicine. A community has some well-established virtues, but critical reflection of these values may stimulate changes. The main virtues endorsed by healthcare personnel in Mexico are 'trustworthiness', 'intellectual honesty' and 'beneficence', followed by 'fortitude', 'compassion' and 'courage'. The CME intervention had a significant strengthening effect on these virtues among the participants (Figures 4 and 8).

## Discussion

We have thus shown experimentally that the two paradigms, EBM and VBM, can be converted into an EBM-VBM binomial through CME intervention. (Figures 3, 4, 5, 6, 7, 8 and Tables 2 and 3).

Our results demonstrate that the healthcare personnel participating in a CME intervention in clinical ethics improved high-order values Openness to Change and Self-Transcendence, as observed through a concurrent triangulation approach (Figures 6A, Band 7A-C and Table 2). Quantitative analysis showed that even if core values, such as honesty and respect, remained unchanged after educational intervention, other important values for the healthcare professional-patient relationship were changed (for example, justice, autonomy, love and charity). Moreover, the representations of values found in the qualitative analysis are compatible with the preceding results (compassion, justice, beneficence). In both cases, deontological and aretological values are susceptible to change in such a way as to fulfil the healing aims of medicine [2].

Ethical discernment is a daunting task from the perspective of integral ethics, which seeks the good of the patient by integrating the three characteristics of action: as a moral agent, as a process to be evaluated from the perspective of rights and duties, and with respect to the consequences of the action. This approach ensures that healthcare professionals exercise their hierarchy of values (Figure 4 and 8).

Following Lozano, dilemmas can arise from different areas; different values and different kinds of ethics take precedence. Values consider the good that is to be achieved through a practice. However, when several of these values are in conflict and a decision cannot be reached, virtue becomes central in solving an ethical problem; the character, practical wisdom and experience of the practitioner are important resources for ethical deliberation (Figure 8) [7,9,12,14,42,43].

CME must focus on four areas to strengthen the EBM-VBM binomial: The first area is extensive knowledge of the pathophysiology of disease and availability of real therapeutic alternatives (EBM). The second area is knowledge and awareness of values (VBM). The third one is development of an ability to analyze and discern ethical dilemmas (VBM), and the fourth area is communication skills (VBM) [44,45].

In this study, we validated our instrument (a value hierarchy in clinical practice) and identified the opportunity for CME intervention. Figure 3 demonstrates that participants recognized that, to act fully as providers of medical assistance, advisors and educators, the most important values are honesty, respect and autonomy. These values are similar to those enumerated in the literature [46]. These specific roles lend a multi-dimensional character to the patient-healthcare professional relationship. Our results (Table 3) show that the most established role in this group of professionals is that of a provider of medical assistance; however, the CME intervention successfully developed bridges and networks to improve the practitioners' performance in the role of educator and adviser.

A notable outcome of our CME clinical ethics course is that it created awareness among physicians and healthcare professionals of how their decisions are made and the values that are at stake (Figures 3, 4, 5, 6, 7, 8). Moreover, the intellectual exercise involving several ethical perspectives ensured that healthcare professionals deliberated in a comprehensive and thoughtful manner (Figures 3, 4, 5, 6, 7, 8).

We propose that an integral approach to ethics similar to the one described by Polo [40] is the solution to the increasing number of ethical dilemmas in medicine. Such an approach considers three features of ethical discernment: 1) The best decision is an integrated one that does not spring from isolated principles but one that allows the moral agent to consider due processes in a given context, as well as his/her own virtues in determining a course of action [14]. Therefore, deontological, aretological and utilitarian criteria may overlap in decision making.

2) These criteria emphasize different issues involved in an ethical dilemma. A deontological criterion focuses on the fulfilment of general and comprehensive rules. A focus on virtue is more capable of addressing the immediate context. Moreover, a value such as efficiency should not be neglected in the healthcare professions. However, the specific configuration of the dilemma and the moral character of those involved will reveal which criteria should be prioritized in a process of wide reflexive equilibrium [47,48].

3) The most important criterion for decision making is the respect for the universal trait of human dignity, even if those who address this dilemma conceive of it in different manners. Dignity supports the individual in acting conscientiously and making his/her own life choices [49,50].

It is of the utmost importance to keep in mind the primacy of those affected by the decisions, ethical values and discernment of healthcare personnel. However, the decisions made by healthcare personnel not only affect patients but also the decision-makers themselves. These decisions determine what kind of person and professional one becomes, how sensitive one is towards others, and how attentive one is towards one's own needs. The best decisions justifiably solve a dilemma by integrating different ethical theories related to human actions. An integrated ethics approach is essential in medicine because medicine is concerned with the person and his/her interests, needs, vocation, virtues and transcendence.

In a nutshell, we can say that the CME intervention favors the creation of networks between EBM and VBM from a philosophical, epistemic, axiological and practical point of view. From the philosophical point of view, we were able to recover the ends of medical practice, namely: healing, curing and caring, the recognition of the person as central to healthcare. This is evident in Table 3 where after the CME intervention; the representations and beliefs of the healthcare personnel were modified, and exhibited a more thorough understanding of the philosophy of medicine. From the epistemic point of view, the epistemic values that are related with medical attention were strengthened. Simultaneously, we perceived increased knowledge related to integral ethics. This can be seen in Table 3 and Figures 3, 4, 5, 6, 7, 8, where the physicians' roles were more balanced. The promotion of personal growth through the exercise of values, such as autonomy, love, justice and freedom, could be seen. These values were rediscovered and used to improve the patient-healthcare personnel relationship. Both the quantitative (Figures 3, 4, 5, 6), and the qualitative (Figures 7 and 8, and Table 3) analyses show the construction of links between EBM and VBM. From a practical perspective, tools for ethical discernment were provided, discursive spaces to reflect and critically analyze ethical dilemmas in clinical practice were created.

In ethical discernment, the use of clinical vignettes to picture situations with moral dilemmas proved useful. In these exercises autonomy stands out, it was a value previously overlooked and it became relevant for the healthcare personnel after the CME intervention. Additionally, ethical committees as guarantors of the humanization of healthcare were consolidated, while the promotion of a professional environment directed by academic, ethical and social excellence was encouraged. Strengthening values-based medicine automatically strengthens the EBM-VBM binomial given that they become more balanced. This is clearly shown in the present study. We are looking forward to continuing with the next phase of this project, which involves carrying out participant observation of the active healthcare personnel in medical units, in order to follow up on these results in the long term. This study is one of the first to explore the axiology of clinical practice. Different values and representations may be found depending on the studied population; however, we consider that the empirical method used to explore the representations of these professionals opens a window of opportunity for CME insofar as it strengthens the already-existing values among healthcare personnel; and at the same time, it promotes values that are missing but essential to an effective patient-healthcare personnel relationship. One dimension of medical responsibility involves being attentive to the values that need to be exercised. López Quintás [51] explains that this attentiveness requires certain value-sensitivity: an ability to discover and recognize the fertility that values have in our lives, when they offer authentic possibilities for personal growth.

## Conclusions

This is the first endeavor to empirically investigate the axiological foundations of healthcare professionals working in Mexico. It has long been known that values education is one of the most effective methods to meet the challenge of providing high-quality care to populations and improving the patient-healthcare professional relationship [1,11,44]. Even if different configurations of these values operate in each medical environment, by identifying the values already held in high esteem and those that need to be encouraged, we are certain that these benefits can be extended globally to every level of care.

For CME design purposes, we successfully engineered networks between EBM and VBM. Using the combined approach of cross-functional design, online technology, motivational videos, pictures and real-time decision-making, these networks identified the advantages of both paradigms. Perhaps the CME methods used in this study will encourage the humanization of medicine through routes not open to traditional CME methods, thus potentially allowing access to more efficient CME solutions, as in the example presented here.

It has long been speculated that CME in clinical ethics in real time may be a useful platform for engineering novel networks between EBM and VBM. Our strategy of cross-functional CME in clinical ethics may be of broad application in achieving high-quality care.

## Abbreviations

CASC: capacity to appraise situations and consequences; CME: continuing medical education; C/N: compliance with the norm; CO: conservation; CVs: clinical vignettes; EBM: evidence-based medicine; EVAT: Escala de Valores hacia el Trabajo, Spanish for work values scale; IMSS: Mexican Institute of Social Security; OC: openness to change; OM: order mean; SE: self-enhancement; SSIs: semi-structured interviews; ST: self-transcendence; VBM: values-based medicine

## Competing interests

The authors declare that they have no competing interests.

## Authors' contributions

MMAB and NFAB conceived and designed the present study, while MMAB, MTAO, SQV, JM, RND and ACD collected and assembled the data. MMAB, NFAB, AL, IMM, AdH, CM, SQV, JM, RND, FVG, RV, EC, PS and JMMA contributed to analyzing and interpreting the data. MMAB, NFAB, AL, IMM and AdH drafted the article. MMAB, NFAB, AL, AdH, FS, PS, CM, JMMA, FVG, JG, SIA, MTAO, JAA, OMG, ARF and JKR revised the article for important intellectual content. All authors gave their final approval.

## End notes

^a^Of these two paradigms, EBM appears to predominate: Medline displays 49,491 EBM articles versus 1,701 articles addressing Humanistic Medicine, Patient-Centered Medicine or VBM. ^b^Axiology is the philosophical discipline that studies values and the phenomena surrounding them. ^c^De Hoyos A, Nava-Diosdado M, Mendez J, Ricco S, Serrano C, Macias-Ojeda C, Cisneros H, Bialostozky D, Altamirano-Bustamante N, Altamirano-Bustamante MM: **Cardiovascular medicine at face value: a pilot qualitative study on clinical axiology**. *Philos Ethics Humanit Med *2013 (accepted). ^d ^ATLAS.ti Scientific Software Development GmbH, Berlín, Germany.

## Pre-publication history

The pre-publication history for this paper can be accessed here:

http://www.biomedcentral.com/1741-7015/11/39/prepub
